# Pathogen metadata platform: software for accessing and analyzing pathogen strain information

**DOI:** 10.1186/s12859-016-1231-2

**Published:** 2016-09-15

**Authors:** Wenling E. Chang, Matthew W. Peterson, Christopher D. Garay, Tonia Korves

**Affiliations:** 1 Data Analytics Department, The MITRE Corporation, 2280 Historic Decatur Rd, San Diego, CA 92106 USA; 2 Data Analytics Department, The MITRE Corporation, 202 Burlington Rd, Bedford, MA 01730 USA

**Keywords:** Pathogen, Metadata, BioSample, LabKey, Biosurveillance, Geocoding, PostgreSQL, Java

## Abstract

**Background:**

Pathogen metadata includes information about where and when a pathogen was collected and the type of environment it came from. Along with genomic nucleotide sequence data, this metadata is growing rapidly and becoming a valuable resource not only for research but for biosurveillance and public health. However, current freely available tools for analyzing this data are geared towards bioinformaticians and/or do not provide summaries and visualizations needed to readily interpret results.

**Results:**

We designed a platform to easily access and summarize data about pathogen samples. The software includes a PostgreSQL database that captures metadata useful for disease outbreak investigations, and scripts for downloading and parsing data from NCBI BioSample and BioProject into the database. The software provides a user interface to query metadata and obtain standardized results in an exportable, tab-delimited format. To visually summarize results, the user interface provides a 2D histogram for user-selected metadata types and mapping of geolocated entries. The software is built on the LabKey data platform, an open-source data management platform, which enables developers to add functionalities. We demonstrate the use of the software in querying for a pathogen serovar and for genome sequence identifiers.

**Conclusions:**

This software enables users to create a local database for pathogen metadata, populate it with data from NCBI, easily query the data, and obtain visual summaries. Some of the components, such as the database, are modular and can be incorporated into other data platforms. The source code is freely available for download at https://github.com/wchangmitre/bioattribution.

**Electronic supplementary material:**

The online version of this article (doi:10.1186/s12859-016-1231-2) contains supplementary material, which is available to authorized users.

## Background

With advances in DNA sequencing technology, whole genome sequencing of pathogen strains from disease outbreaks is becoming routine. These advances are resulting in enormous growth in the amount of publicly available pathogen nucleotide sequence data. One critical component of this data is high-quality metadata about biological samples. This metadata includes information about where the sample originated and the sample’s phenotypic properties. These types of features include, but are not limited to, geolocation data, isolation source, collection date, the organization performing collection, sample and strain names, and drug or vaccine resistance information. Pathogen sample metadata presents new opportunities for diagnostic and treatment discovery, biosurveillance, and public health investigations. In order for many of these opportunities to be realized, pathogen metadata needs to be made easily accessible to those beyond the bioinformatics community.

There has been significant growth in the capture and sharing of pathogen metadata. The Genomic Standards Consortium (GSC) has developed a set of “Minimal Information about any Sequence” (MIxS) checklists for genomes (MIGS), including checklists specifically for pathogen samples [1, 2]. Recently, a consortium of pathogen-sequencing institutions created a new metadata standard for pathogens, called the GSCID/BRC (Genome Sequencing Centers for Infectious Diseases and Bioinformatics Resource Centers) Project and Sample Application Standard [3]. Repositories for pathogen metadata have also been created. The National Center for Biotechnology Information (NCBI) maintains the BioSample and BioProject databases [4], which contain metadata about biological samples and projects, respectively. This data is typically submitted by investigators in concert with submission of nucleotide sequence data. BioSample and BioProject databases exchange data with their European and Japanese counterparts [5]. The Pathosystems Resource Integration Center (PATRIC) and the Virus Pathogen Database and Analysis Resource (ViPR) also provide standardized metadata for some pathogenic bacterial and viral genomes, respectively [6, 7]. The Genomes Online Database (GOLD) [8], developed at the Department of Energy Joint Genomes Institute, is a manually curated warehouse of metadata about sequencing experiments following the MIxS standards. There have also been a number of tools developed to query and retrieve this metadata. The Entrez system at the NCBI [9] provides an interface for searching and filtering query results, and tools such as BioPython [10], BioPerl [11], and BioJava [11] provide functionality for interfacing with these web services. SRAdb enables access to the Sequence Read Archive metadata using R [12].

For biosurveillance and public health endeavors, there are advantages to hosting an independent data platform incorporating publicly available pathogen metadata. In particular, this allows institutions to integrate other data critical for the mission and analyze it in concert with NCBI sample data. For biosurveillance and public health, the joint analysis of pathogen metadata and epidemiological data will be particularly important. Institutions may also have additional pathogen sample data not associated with genomes, or sample data an institution does not want to make public to be analyzed in concert with publicly available data. Furthermore, a separate database allows institutions to customize the database by further standardizing data or adding data fields and tables.

This manuscript describes a web server application designed to make pathogen metadata readily accessible to biologists, biosurveillance analysts, and public health investigators without requiring computer programming. The software includes a database for the capture of pathogen metadata, scripts to populate the database with metadata from NCBI BioSample and BioProject and a user interface to query, obtain standardized metadata, and visually summarize results.

## Implementation

### The sample metadata database schema

The sample metadata database is a PostgreSQL database designed to store information about pathogen samples. The schema captures information types that occur in BioSample and BioProject pathogen submissions, and uses many terms from MIxS. The tables in the database are summarized in Table 1. Additional file 1: Figure S1 shows the relationships between these tables, and the database is documented in detail in the *BioAttDB_Documentation.pdf* file provided with the software.

**Table 1:** Overview of the tables in the sample metadata database

Database table	Content
Sample	Identity of a sample, including strain name, serovar, and submission date
Collection	Where, when and what type of environment the sample was collected from
Human_Host	Information about the human host for clinical samples, such as age and gender
Non_Human_Host	Information about non-human hosts for environmental samples
Study_Method	Methods used for obtaining and identifying a sample
Project	Information about the project associated with the strain
Project_Sample	Links projects to samples
Owner	Information about the organization that submitted the information about a strain
Collection_Owner	Information about the organization that collected the strain
Project_Publication	Links a sample to publications by PubMed Identifier
Cross_Reference	Stores source and id pairs for documents and databases that reference a sample

### Scripts to import, parse, and standardize metadata from NCBI

The import of NCBI metadata into the metadata database is handled in four steps. In the first step, performed by the *DataDownload.sh* script, the BioProject and BioSample XML files are downloaded from the NCBI FTP server. Next, the *DataSplit.sh* script splits the single XML file provided by NCBI into multiple files containing a subset of the nodes relevant to the database schema for more efficient parsing. Parsing is performed by a Java program, which uses a document object model (DOM) parser to map the XML files to Java classes, create tables, and load the data into the database. When the BioProject and BioSample XML schemas are changed by NCBI, the parser code will need to be updated to reflect the changes. The *DataMapping.sh* script calls the parser and pre-parses the XML files to create a mapping between BioProject and BioSample files. Finally, the *DataUpdate.sh* calls the parser twice – once to create the database, and once to load the data into the database.

### LabKey module for database query and visualization

LabKey Server [13] is a data management platform designed for biological data. It is a modular, web-based Java application allowing users to create database schemas, queries, forms, and visualizations in support of research. Rather than requiring the user to load the data into LabKey’s schema, we have chosen to interface with the Metadata Database. This allows investigators who may be using another system to interface with the database without having to use LabKey. For those using LabKey, the module provides a simple interface to query the metadata database, and make the data available via the LabKey APIs. The interface and query logic is written in HTML and JavaScript, and is easily extendable by the end user. Once a query is performed, results are displayed in a table and can be filtered, visualized, and exported using the capabilities built into LabKey.

In addition to the built-in table and graph views from LabKey, the module adds the ability to summarize the results of a query in the form of a 2D histogram. The visualization, which is built using D3.js [14], creates a two-dimensional histogram using two variables selected by the user. The visualization is interactive, allowing the user to mouse over to see the exact count for any given combination.

In addition to the 2D histogram view, the software provides functionality to geocode based on any column in a List (LabKey’s user-created database tables) and display the results on a map. In this distribution, the geocoding and mapping is performed using a Google Maps API (https://developers.google.com/maps/), though this could be changed by the end user to use a geospatial analysis package of their choice.

## Results and discussion

In this section, we highlight two examples showing how the Pathogen Metadata Platform can be used in the investigation of disease outbreaks. In these examples, the database has been populated with data from NCBI on October 27, 2014. Time to populate the database will depend on the current size of BioSample and BioProject, connection speed, parameters used for splitting, and processor speed. On our system, upload time for the database in May 2016, with size 4.7 GB, was less than 16 h.

### Identifying and Summarizing Strain Data for a Pathogen Species

**Fig. 1:**
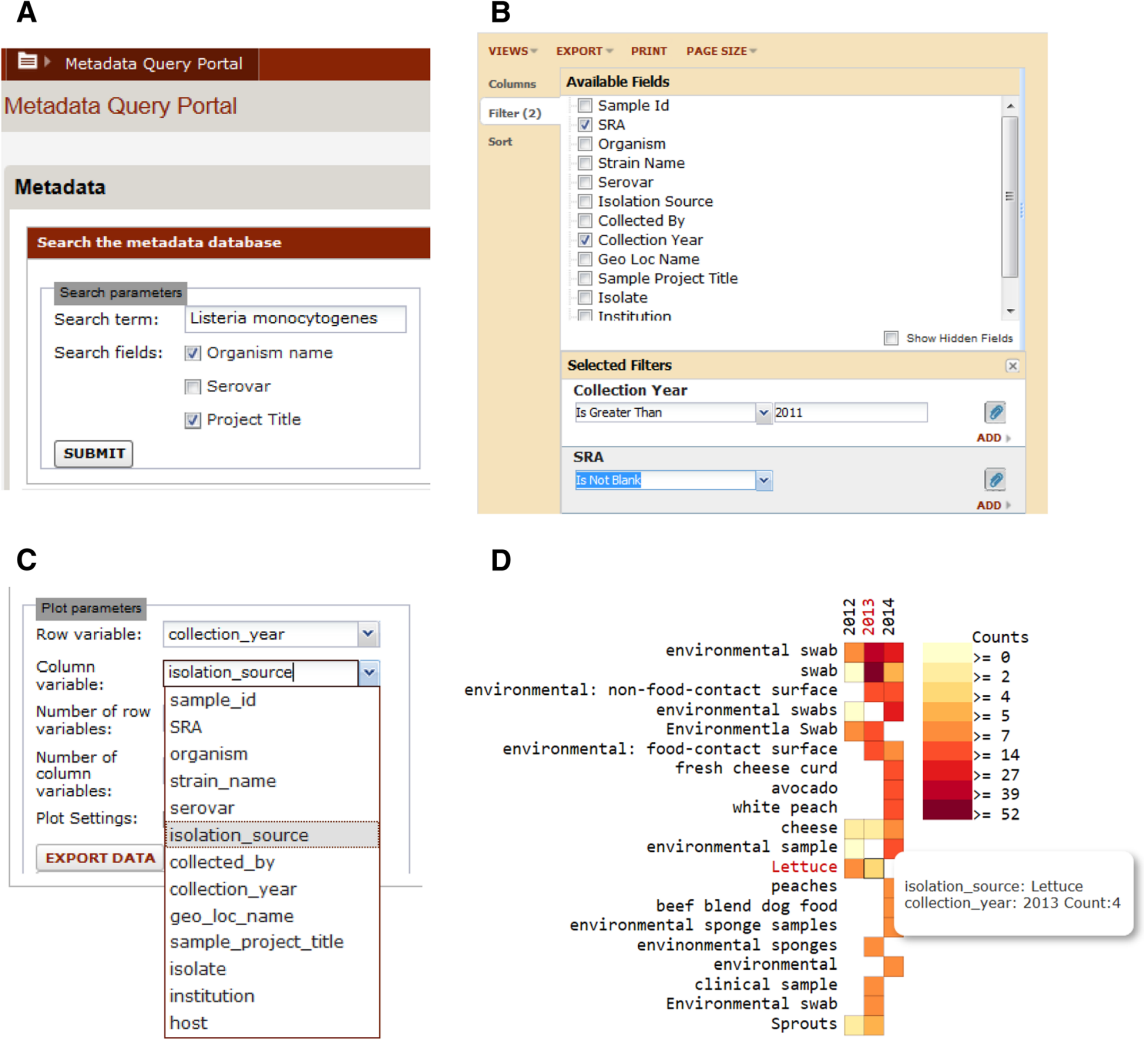
Obtaining information about *Listeria* strains using the Pathogen Metadata Platform. **a** Querying for a pathogen name. **b** Filtering query results. **c** Selecting metadata types for a 2D histogram. **d** 2D histogram of counts for two metadata types

In this example, there is a new disease outbreak and an investigator wants to determine whether there have been recent outbreaks that may be related. The investigator performs a search on the pathogen name using the basic query interface. Figure 1a shows a search for samples containing data from *Listeria monocytogenes.* The results are returned in the form of a LabKey table view, which contains information about the samples, including relevant metadata such as strain name, isolation source, collection date, serovar, as well as a reference to the accession number in the NCBI Sequence Read Archive (SRA). This table is then filtered to include only samples collected within the past three years, as shown in Fig. 1b. The table can be exported for use in a bioinformatics analysis pipeline in order to, for example, identify which strains are most closely related to the outbreak strain. Finally, the filtered data is summarized via the 2D histogram view. Figure 1c-d shows the creation of a 2D histogram showing the number of samples collected across years and isolation sources for insight into potential types of sources of the outbreak.

### Obtaining and visualizing information about closely related pathogen strains

In this example, investigators have sequenced a pathogen sample from a patient and performed phylogenetic analyses using RAxML [15], phylogenetic software that uses a maximum likelihood approach. This identified 22 *Salmonella enterica* serovar Typhimurium genomes from NCBI that are closely related to the patient’s strain. The investigator wants to know where and what type of environments these closely related strains came from.

**Fig. 2:**
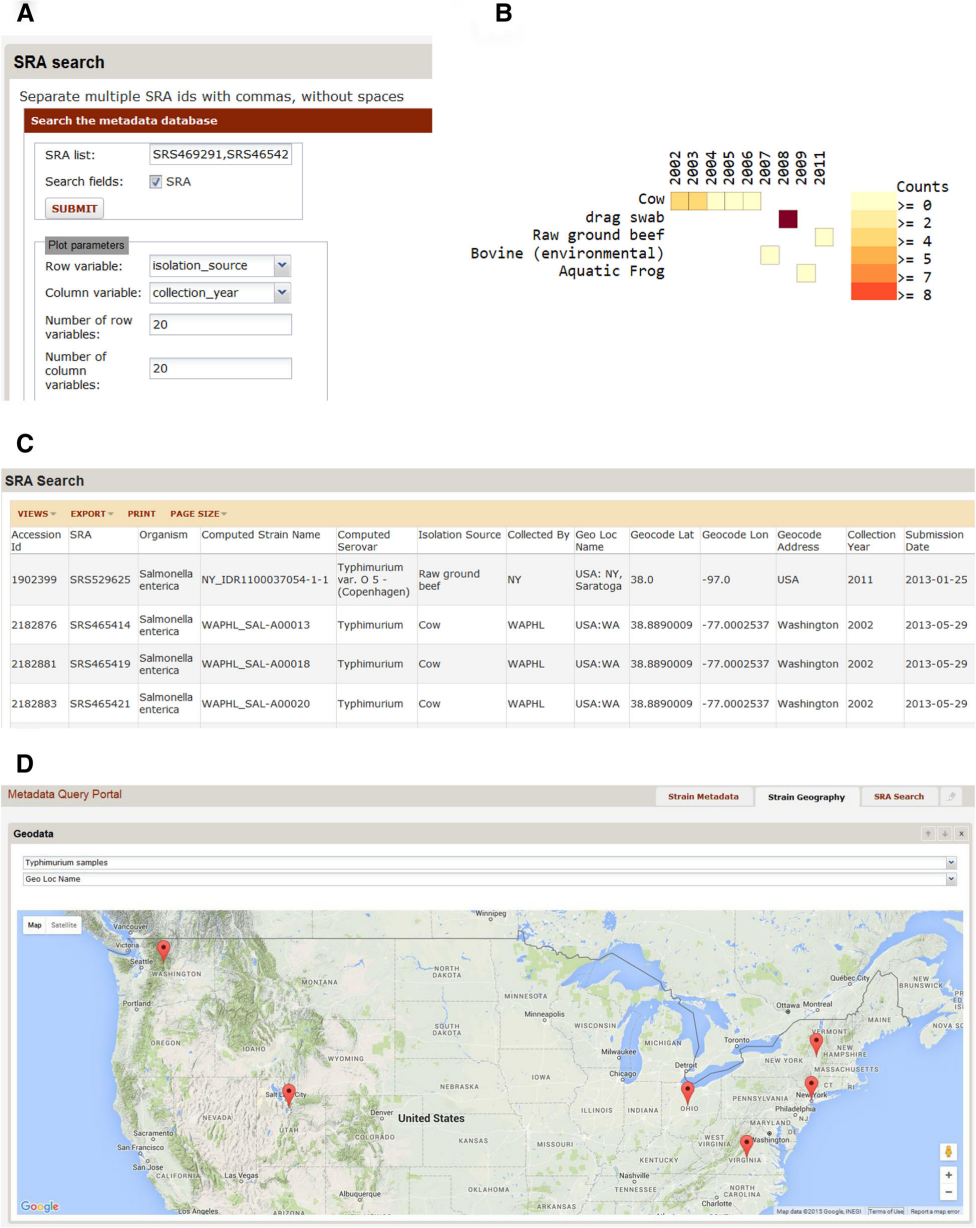
Obtaining and visualizing metadata for *Salmonella* strains. **a** Querying for a set of sequence read archive ids. **b** 2D histogram of counts for isolation source and year. **c** Table of query results. **d** Map of collection locations of the strains

Information about these strains can be obtained by using the SRA Search form within the LabKey module. SRA identifiers are entered as a comma-separated list (Fig. 2a) and are returned as a LabKey table (Fig. 2c). This table is then filtered and a 2D histogram summarizing isolation sources and collection years is created as in the previous example (Fig. 2b).

The collection locations of these strains are then mapped. To do this, the table of results is exported as a LabKey list. The “Strain Geography” tab within the LabKey Module allows the user to select this list, along with the column containing the location information to be passed to the geocoder. A map is then presented, with each strain with a location returned by the geocoder displayed as a point on the map (Fig. 2d). Here, we see that the majority of the closely-related strains found within the United States are located in the northeast.

### Relationship to other resources

The Pathogen Metadata Platform offers a few advantages relative to other currently available resources. First, once installed, the platform provides an easy way to query and obtain tables of standardized metadata. In this respect is it similar to capabilities offered in ViPR for some virus genomes [6], and in PATRIC for assembled bacterial genomes [7], but provides access to all sample entries in BioSample including for the growing number associated with unassembled genomic data. Second, the platform integrates mapping of geographical locations for genomes from a large database. Available software for mapping geolocations of pathogen genomes includes Supramap, which superimposes phylogenies onto a map [16], and GoMap, which is currently implemented to map HIV strains with drug resistance mutation information [17]. Unlike these, the Pathogen Metadata Platform links mapping with all samples from BioSample, though without a DNA analysis component. In addition, the platform provides interactive 2D histograms to show the variables most strongly associated with the queried pathogen, such as types of environments the pathogen is frequently collected from. Interactive summary figures for pathogen genome metadata have not been incorporated into other webserver applications yet, but provide a way to understand pathogen context quickly, especially when there are large numbers of genomes per species.

## Conclusions

The Pathogen Metadata Platform provides functionalities for parsing and loading metadata from NCBI into a relational schema, as well as query and visualization capabilities. This open-source software is modular, such that some components can be individually incorporated into other platforms and modified for specific purposes. For example, the metadata database could be used with other software, and data from sources other than NCBI can be added to it. In addition, the software is extensible, and the LabKey platform provides the opportunity to develop modules for additional analyses. We believe this software will be particularly useful as a complement to DNA analyses, as it has been in our own research. The platform could be paired with easy-to-use DNA analysis software that assesses the relatedness of pathogen strains to enable biosurveillance and public health investigations.

## Availability and requirements

**Project Name**: Pathogen Metadata Platform

**Project Home Page**: https://github.com/wchangmitre/bioattribution

**Operating system**: Linux

**Programming Environment**: Java, SQL

**Requirements**: A working installation of LabKey Server and PostgreSQL database server

**License**: Apache License

## Additional file

Additional file 1: Figure S1. Metadata database schema. (PPTX 59 kb)

## Abbreviations

DOM: Document object model
GoMap: Geogenomic mutational atlas of pathogens
GSC: Genomic standards consortium
MIGS: Minimum information about a genome sequence
MIMARKS: Minimum information about a marker gene sequence
MIMS: Minimum information about a metagenome sequence
MIxS: Minimum information about any sequence
NCBI: National center for biotechnology information
SRA: Sequence read archive
UI: User interface
ViPR: Virus pathogen database and analysis resource
