# Supplementary figures and images for: Pathogen metadata platform: software for accessing and analyzing pathogen strain information
Source: BMC Bioinformatics. 2016 Sep 15;17:379. doi: 10.1186/s12859-016-1231-2 (PMC5025631; doi:10.1186/s12859-016-1231-2)

## Slide 1
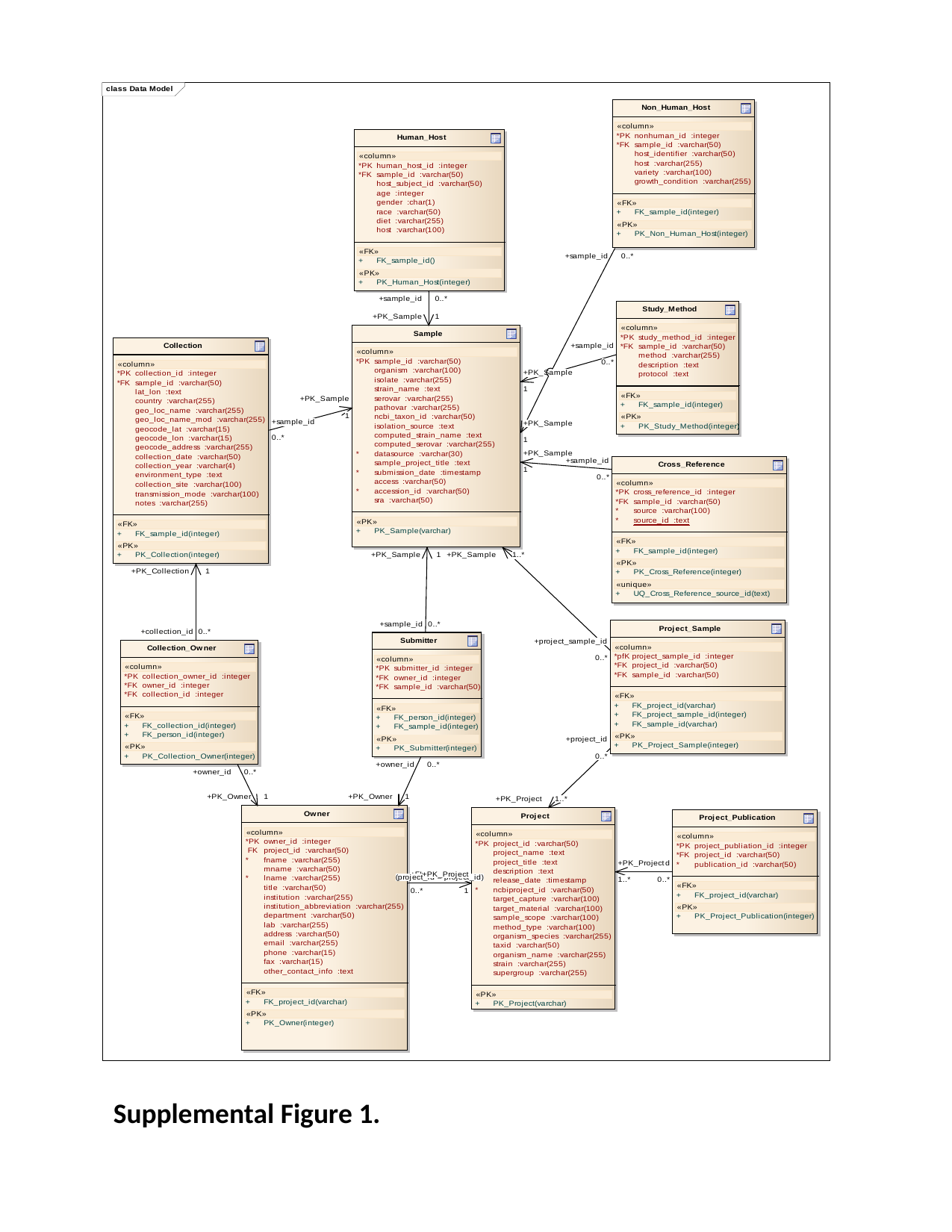

Supplemental Figure 1.

Supplement: Additional file 1: Figure S1. — Metadata database schema. (PPTX 59 kb) [file 12859_2016_1231_MOESM1_ESM.pptx]
